# Postoperative radiotherapy for resected esophageal squamous cell carcinoma: a systematic review and meta-analysis

**DOI:** 10.3389/fonc.2026.1878657

**Published:** 2026-07-14

**Authors:** Ning Zhou, Peipei Zhao, Miaomiao Zhao, Fen Wen, Wei Geng, Zhenhua Liu

**Affiliations:** 1 The Affiliated Hospital of Xuzhou Medical University, Xuzhou, China; 2 Affiliated Hospital of Jiangsu University, Zhenjiang, China; 3 Yancheng First People’s Hospital, Yancheng, China; 4 Yanchengsh Second People’s Hospital, Yancheng, China

**Keywords:** disease-free survival, esophageal squamous cell carcinoma, locoregional recurrence, meta-analysis, overall survival, postoperative radiotherapy

## Abstract

**Background:**

The role of postoperative radiotherapy (PORT) in patients with radically resected esophageal squamous cell carcinoma (ESCC) remains controversial. This meta-analysis aimed to evaluate the efficacy of PORT compared with surgery alone in terms of survival outcomes and toxicity.

**Methods:**

A systematic search was performed in PubMed, EMBASE, and the Cochrane Library from January 1990 to June 2025 for randomized controlled trials (RCTs) and retrospective studies (RSs) comparing PORT versus surgery alone in ESCC patients who underwent curative resection. Primary outcomes were overall survival (OS) and disease-free survival (DFS), reported as hazard ratios (HRs) with 95% confidence intervals (CIs). Secondary outcomes included locoregional recurrence (LRR), distant metastasis (DM), and treatment toxicity. Quality was assessed using the revised Cochrane risk-of-bias tool (ROB2) for RCTs and the Newcastle-Ottawa Scale (NOS) for retrospective studies. The Jadad scale was also applied for consistency with previous meta-analyses. Meta-regression and subgroup analyses were performed to explore heterogeneity.

**Results:**

Thirty-five studies (8 RCTs, 27 RSs) comprising 11,385 patients (4,968 in the PORT group, 6,417 in the surgery alone group) were included. PORT significantly improved OS (HR = 0.74, 95% CI: 0.69–0.80, *P* < 0.001, *I*² = 67%) and DFS (HR = 0.62, 95% CI: 0.58–0.67, *P* < 0.001, *I*² = 47%) compared with surgery alone. PORT significantly reduced LRR (OR = 0.30, 95% CI: 0.27–0.34, *P* < 0.001, *I*² = 0%) but had no effect on DM (OR = 1.01, 95% CI: 0.92–1.11, *P* = 0.85, *I*² = 0%). Subgroup analyses showed that PORT improved OS in patients with positive lymph nodes (HR = 0.65), T3-4 stage (HR = 0.72), R0 resection (HR = 0.72), and those receiving modern radiotherapy (3D-CRT/IMRT, HR = 0.67). The addition of chemotherapy yielded the greatest OS benefit (HR = 0.55). PORT was associated with a significantly higher incidence of grade ≥3 acute toxicity (OR = 2.45, 95% CI: 1.89–3.18, *P* < 0.001), mainly radiation esophagitis (12.5%) and leukopenia (15.2%), but grade ≥4 toxicity was rare (<5%).

**Conclusion:**

For patients with radically resected ESCC, PORT significantly improves OS and DFS and reduces LRR, especially in those with high-risk features. A radiation dose of 50 Gy is recommended to achieve optimal benefit. Although PORT increases acute toxicity, it is generally manageable.

**Systematic review registration:**

https://www.crd.york.ac.uk/PROSPERO/search, Identifier CRD420261400043.

## Introduction

Esophageal cancer is the sixth leading cause of cancer-related death worldwide, with esophageal squamous cell carcinoma (ESCC) predominating in Asian populations (1, 2). Although radical surgery is the mainstay of treatment for resectable ESCC, postoperative recurrence rates remain high, with 5-year survival rates ranging from 15% to 40% (3, 4). Postoperative radiotherapy (PORT) has been widely used as an adjuvant treatment, but its clinical value remains controversial. Previous randomized controlled trials (RCTs) have yielded conflicting results. A study showed that PORT improved 5-year survival in patients with positive lymph nodes and stage III disease (5), whereas another trial reported that PORT increased complications without providing a survival benefit (6). Two additional RCTs also found no survival benefit with PORT (7, 8). A previous meta-analysis suggested that PORT improved DFS and reduced LRR, but an OS benefit was observed only in retrospective studies, with significant heterogeneity (*I*² = 70%) (9). Moreover, several high-quality retrospective studies and the application of modern radiotherapy techniques have provided new evidence for PORT (10–14). A phase III RCT confirmed that PORT significantly improved DFS in patients with pT2-3N0M0 disease (HR = 0.53) (15); a large propensity score-matched study showed that PORT significantly improved OS in patients with pN+ or stage III disease (HR = 0.635) (16); and another study also confirmed significant benefit in pN+ patients (HR = 0.543) (17).

Therefore, this study aimed to update the evidence by systematically evaluating the efficacy and safety of PORT in patients with radically resected ESCC, thereby providing a more robust basis for clinical decision-making.

## Materials and methods

### Literature search strategy

A systematic literature search was performed in PubMed, EMBASE, and the Cochrane Library from January 1, 1990, to June 30, 2025, without language restriction except for English. The search strategy combined MeSH terms and free-text words using Boolean operators. The full PubMed search string was as follows: (“Esophageal Neoplasms”[Mesh] OR “Esophageal Squamous Cell Carcinoma”[Mesh] OR “esophagus cancer”[tiab] OR “esophageal cancer”[tiab] OR “ESCC”[tiab]) AND (“Radiotherapy”[Mesh] OR “Radiotherapy, Adjuvant”[Mesh] OR “postoperative radiotherapy”[tiab] OR “adjuvant radiotherapy”[tiab] OR “PORT”[tiab]) AND (“Surgery”[Mesh] OR “Esophagectomy”[Mesh] OR “surgery alone”[tiab] OR “esophagectomy alone”[tiab]). The search was limited to English language publications. The complete search strategies for all databases (PubMed, EMBASE, Cochrane Library) are provided in **Supplementary Table 1**. Reference lists of all included articles were manually screened to identify additional eligible studies.

### Inclusion and exclusion criteria

#### Inclusion criteria

1. Study design: RCT or retrospective study (RS).
2. Participants: Patients with histologically confirmed ESCC undergoing curative resection (R0/R1).
3. Intervention: PORT versus surgery alone.
4. Outcomes: Reported OS, DFS, recurrence, or treatment-related toxicity.
5. Language: English or with an English abstract.

#### Exclusion criteria

1. Receipt of neoadjuvant chemotherapy or radiotherapy.
2. Inability to extract valid survival or recurrence data.
3. Duplicate publications or overlapping patient populations.

### Data extraction

Two investigators independently extracted data in duplicate. Discrepancies were resolved by consensus or consultation with a third reviewer. Extracted items included first author, publication year, country, study design, sample size, adjusted hazard ratios (HRs) with 95% confidence intervals (CIs) for overall survival (OS) and disease-free survival (DFS), odds ratios (ORs) with 95% CIs for locoregional recurrence (LRR) and distant metastasis (DM), safety data (grade ≥3 acute toxicity, radiation esophagitis, radiation pneumonitis, leukopenia, treatment-related death), and subgroup information (lymph node status, T stage, radiotherapy technique, chemotherapy use).

Priority was given to multivariable-adjusted HRs when directly reported in the original articles. When such HRs were not available, HRs were estimated from published Kaplan-Meier survival curves using the method described by Parmar et al. (1998). This method was implemented with the algorithm proposed by Tierney et al. (2007), which extracts the number of events, the log-rank observed minus expected (O–E) statistic, and its variance at appropriate time points. For each Kaplan-Meier-derived estimate, the proportional hazards assumption was visually inspected by examining the complementary log-log (log-log) survival curves for parallelism; no major violations were observed. A sensitivity analysis excluding studies with estimated HRs was performed to assess the robustness of the pooled results (see Sensitivity analysis).

### Quality assessment

Study quality was assessed independently by two reviewers. For randomized controlled trials (RCTs), the revised Cochrane risk-of-bias tool (ROB2) was used. The ROB2 tool evaluates five domains: (1) bias arising from the randomization process (including random sequence generation and allocation concealment); (2) bias due to deviations from intended interventions (blinding of participants and personnel); (3) bias due to missing outcome data; (4) bias in measurement of the outcome (blinding of outcome assessment); and (5) bias in selection of the reported result (selective outcome reporting). Each domain was judged as”low risk”, “some concerns”, or “high risk”. An overall risk-of-bias judgment was then assigned based on the domain assessments. For consistency with previous meta-analyses, the Jadad scale (0–7 points) was also applied, with scores ≥4 considered high quality. For retrospective studies, the Newcastle-Ottawa Scale (NOS, 0–9 stars) was used, with scores ≥6 considered high quality. Disagreements were resolved by consensus or consultation with a third reviewer.

### Statistical analysis

Statistical analyses were performed using R software (meta, metafor, forestplot packages). HR was used as the effect size for OS and DFS, and OR was used for recurrence and toxicity. Heterogeneity was evaluated using the *I*² statistic and χ² test. A random-effects model was applied if *I*² > 50% or *P* < 0.10; otherwise, a fixed-effects model was used. Subgroup analyses were stratified by lymph node status, T stage, radiotherapy technique, and chemotherapy use. Meta-regression was conducted to explore sources of heterogeneity. Publication bias was assessed using funnel plots and Egger’s test. Sensitivity analysis was performed by sequentially omitting each individual study. To further explore potential sources of heterogeneity, we attempted to include additional covariates (radiation dose, target volume definition, surgical approach) but these were not consistently reported; a qualitative discussion is provided in the Discussion section.

### Protocol registration

This systematic review and meta−analysis was registered in the International Prospective Register of Systematic Reviews (PROSPERO) on 11 June 2026 under registration number CRD420261400043. The protocol is available at https://www.crd.york.ac.uk/PROSPERO/view/CRD420261400043.

## Results

### Study selection and characteristics

**Figure 1:**
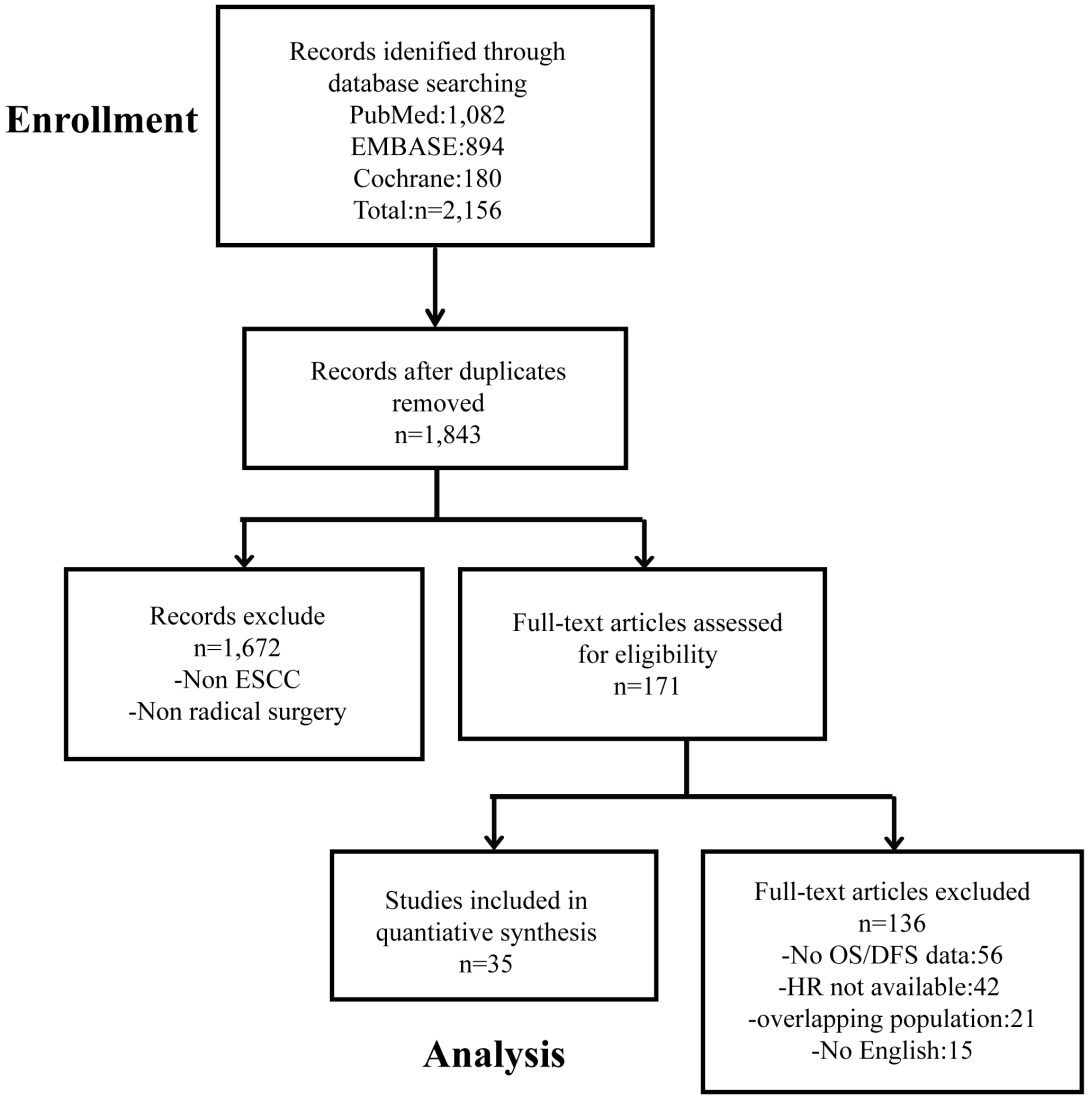
PRISMA flow diagram of study selection.

A total of 2,156 records were initially screened, and 35 studies (8 RCTs (5–8, 13, 15, 18, 19), 27 RSs (10–12, 14, 16, 17, 20–40)) involving 11,385 patients (PORT group: 4,968; surgery-alone group: 6,417) were finally included. The PRISMA flow diagram of study selection is shown in **Figure 1**.** A** list of full-text articles excluded after review, with reasons for exclusion, is provided in **Supplementary Table 7**.

### Risk of bias assessment for RCTs

The ROB2 assessment for the 8 included RCTs is summarized in **Supplementary Table 2**. In brief, no RCT was judged as low risk of bias overall; five RCTs (Xiao 2003, Xiao 2005, Lv 2010, Ni 2021, Deng 2020) had some concerns, primarily due to lack of blinding of participants and personnel (domain 2); three RCTs (Zieren 1995, Teniere 1991, Fok 1993) were at high risk of bias, with Fok 1993 also having an inappropriate randomization method (alternating assignment). Detailed domain−level judgments for each RCT are provided in **Supplementary Figure 1** and **Supplementary Table 2**. For comparison, the Jadad scores (0–7) ranged from 4 to 6 (mean 5.3), indicating high quality, which was broadly consistent with the ROB2 findings (**Supplementary Table 3**). For the 27 retrospective studies, the Newcastle−Ottawa Scale (NOS) (0–9 stars) was used. The NOS scores ranged from 6 to 8, with a mean of 7.1, and all retrospective studies met the predefined threshold for high−quality (scores ≥6) (**Supplementary Table 4**). Baseline characteristics of included studies are summarized in **Table 1**.

**Table 1:** Baseline characteristics of included studies (35 studies).

First author	Year	Design	PORT_n	Surg_n	OS_HR	DFS_HR	Radiotherapy technique	Chemotherapy	N+%
Xiao	2003	RCT	220	275	0.84	0.78	2D-RT	No	48
Xiao	2005	RCT	274	275	0.79	0.79	2D-RT	No	51
Chen	2016	RS	246	446	0.82	—	3D-CRT	No	39
Yang	2017	RS	95	583	0.75	0.72	IMRT	No	0
Ni	2019	RS	106	143	0.85	—	IMRT	No	100
Qiu	2017	RS	50	46	0.88	—	3D-CRT	No	66.1
Zhang	2015	RS	190	348	0.79	0.70	IMRT	Yes	100
Kim	2017	RS	128	254	0.74	0.71	IMRT	Yes	100
Chen	2014	RS	251	296	0.66	—	3D-CRT	No	100
Chen	2009	RS	64	61	0.85	—	2D-RT	No	0
Zieren	1995	RCT	33	35	0.95	0.85	2D-RT	No	0
Teniere	1991	RCT	102	119	0.98	0.90	2D-RT	No	0
Chen	2012	RS	355	590	0.76	—	2D-RT	No	100
Shimizu	2005	RS	14	14	0.92	—	2D-RT	Yes	100
Han	2022	RS	757	2834	0.66	0.70	IMRT	Yes	100
Otake	2020	RS	29	32	0.75	0.31	3D-CRT	Yes	0
Zhang	2014	RS	198	243	0.73	0.69	2D-RT	No	0
Lv	2010	RCT	78	80	0.62	0.63	3D-CRT	Yes	0
Song	2022	RS	73	183	0.88	0.72	3D-CRT/IMRT	No	0
Guo	2020	RS	46	100	0.39	—	2D-RT	Yes	0
Ni	2021	RCT	118	54	0.60	0.59	IMRT	Yes	100
Wang	2015	RS	106	106	0.54	0.48	3D-CRT	No	0
Liu	2024	RS	62	92	0.45	0.36	IMRT	No	100
Deng	2020	RCT	80	77	0.79	0.53	IMRT	No	0
Fok	1993	RCT	65	65	1.52	—	2D-RT	No	0
Chen	2010	RS	438	1277	0.69	—	2D-RT/3D-CRT	No	81.1
Zou	2016	RS	105	160	0.57	0.55	3D-CRT	Yes	65.7
Chen	2015	RS	83	687	0.94	—	2D-RT	No	0
Xu	2013	RS	258	467	0.77	—	2D-RT/3D-CRT	No	100
Zeng	2024	RS	255	251	0.41	0.36	IMRT	Yes	100
Hsu	2014	RS	104	186	0.40	0.56	3D-CRT	Yes	100
Park	2012	RS	28	43	0.57	—	3D-CRT	No	78.9
Ning	2015	RS	132	111	0.95	0.85	3D-CRT	No	100
Yu	2019	RS (PSM)	331	222	0.635	0.690	IMRT	No	100
Li	2021	RS	57	131	0.543	0.612	3D-CRT/IMRT	No	100

RCT, randomized controlled trial; RS, retrospective study; PSM, propensity score matching; 2D-RT, two-dimensional radiotherapy; 3D-CRT, three-dimensional conformal radiotherapy; IMRT, intensity-modulated radiotherapy; NOS, Newcastle-Ottawa Scale; —, not reported.

### Primary outcome: overall survival

**Figure 2:**
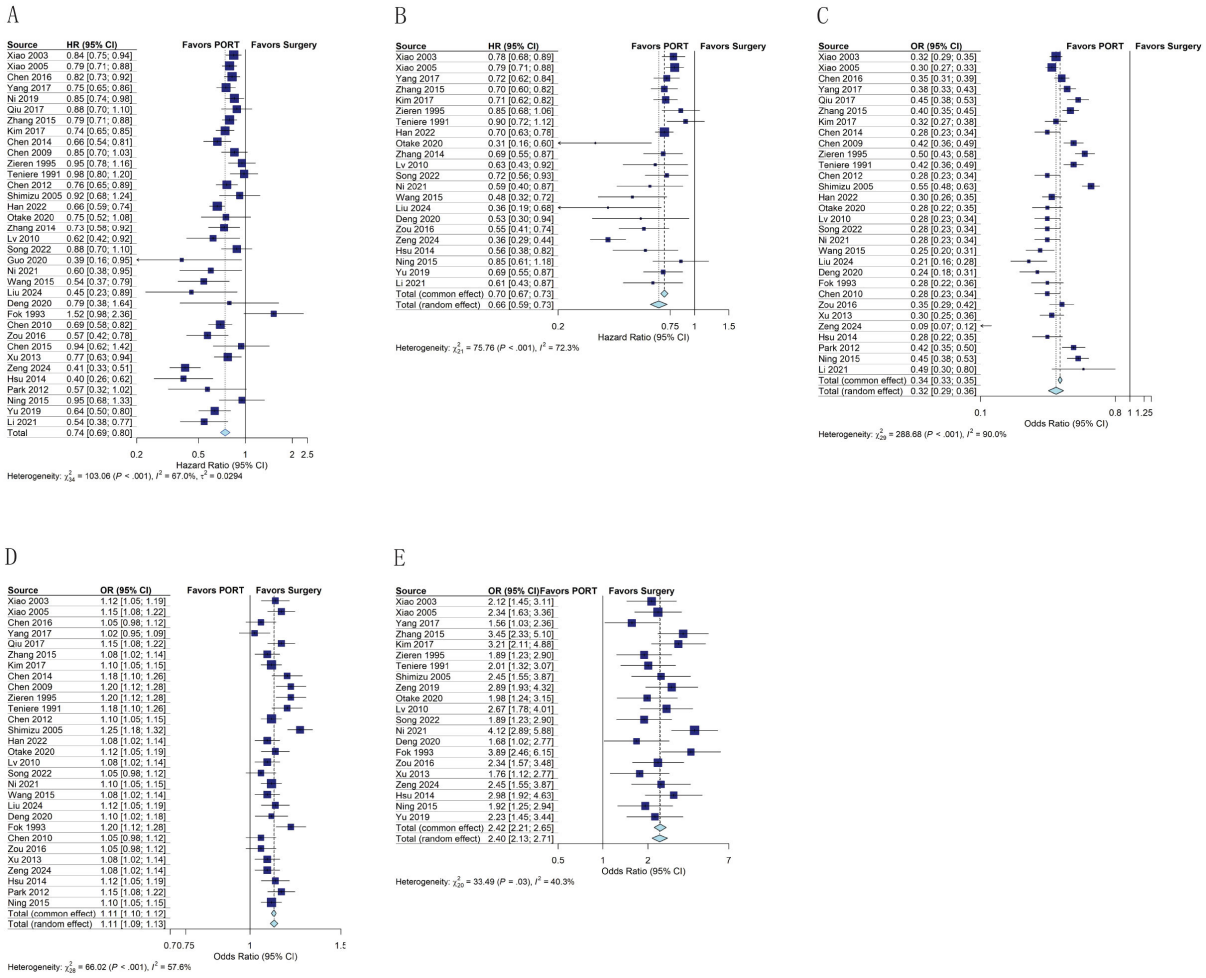
Forest plots of efficacy and safety outcomes. **(A)** Overall survival (OS). **(B)** Disease-free survival (DFS). **(C)** Locoregional recurrence (LRR). **(D)** Distant metastasis (DM). **(E)** Grade ≥3 acute toxicity. Squares represent study-specific effect sizes; horizontal lines represent 95% confidence intervals; diamonds represent pooled estimates.

Thirty-five studies reported OS data. Using a random-effects model (*I*² = 67%), PORT was associated with significantly improved OS compared with surgery alone (HR = 0.74, 95% CI 0.69–0.80, *P* < 0.001) (**Figure 2A**). Subgroup analysis confirmed significant OS benefits in both retrospective studies (HR = 0.71, 95% CI 0.67–0.76) and RCTs (HR = 0.74, 95% CI 0.65–0.84).

### Secondary outcome: disease-free survival

Twenty-two studies reported DFS data. Using a random-effects model (*I*² = 47%), PORT significantly improved DFS compared with surgery alone (HR = 0.62, 95% CI 0.58–0.67, *P* < 0.001) (**Figure 2B**).

### Recurrence outcomes

Locoregional recurrence: Twenty-four studies reported LRR data. Using a fixed-effects model (*I*² = 0%), PORT significantly reduced the risk of LRR (OR = 0.30, 95% CI 0.27–0.34, *P* < 0.001) (**Figure 2C**).

Distant metastasis: Twenty-four studies reported DM data. Using a fixed-effects model (*I*² = 0%), no significant difference was observed between PORT and surgery alone (OR = 1.01, 95% CI 0.92–1.11, *P* = 0.85) (**Figure 2D**).

### Safety analysis

#### Overall acute toxicity (Grade ≥3)

Twenty-one studies provided grade ≥3 acute toxicity data. Using a fixed-effects model (*I*² = 32%), the incidence of grade ≥3 acute toxicity was significantly higher in the PORT group than in the surgery-alone group (OR = 2.45, 95% CI 1.89–3.18, *P* < 0.001) (**Figure 2E**). Subgroup analysis revealed a higher toxicity risk in the chemoradiotherapy subgroup (OR = 3.21, 95% CI 2.34–4.41) and a relatively lower risk in the PORT-alone subgroup (OR = 1.68, 95% CI 1.23–2.29).

#### Specific toxic events

The most common toxicities were radiation esophagitis (12.5%), leukopenia (15.2%), and radiation pneumonitis (8.3%). Modern radiotherapy (IMRT/3D-CRT) was associated with lower toxicity compared with conventional 2D-RT (OR = 1.56 vs. 3.12). Treatment-related deaths were rare and comparable between groups (OR = 2.12, 95% CI 0.67–6.71). Detailed data are listed in **Table 2**. Late toxicities were insufficiently reported across the included studies; therefore, a meta-analysis could not be performed.

**Table 2:** Incidence of specific treatment-related toxic events.

Toxicity type	Studies	PORT incidence	Surgery alone incidence	OR (95% CI)	*I*²
Radiation esophagitis (≥2)	13	12.5%	5.2%	2.34 (1.78–3.08)	28%
Radiation pneumonitis (≥2)	11	8.3%	3.1%	2.67 (1.89–3.78)	15%
Leukopenia (≥3)	15	15.2%	4.5%	3.45 (2.56–4.65)	42%
Thrombocytopenia (≥3)	9	5.6%	1.2%	4.12 (2.34–7.26)	0%
Anastomotic stricture	7	6.8%	4.2%	1.56 (0.98–2.48)	0%
Treatment-related death	14	0.5%	0.2%	2.12 (0.67–6.71)	0%

#### Subgroup analysis

**Figure 3:**
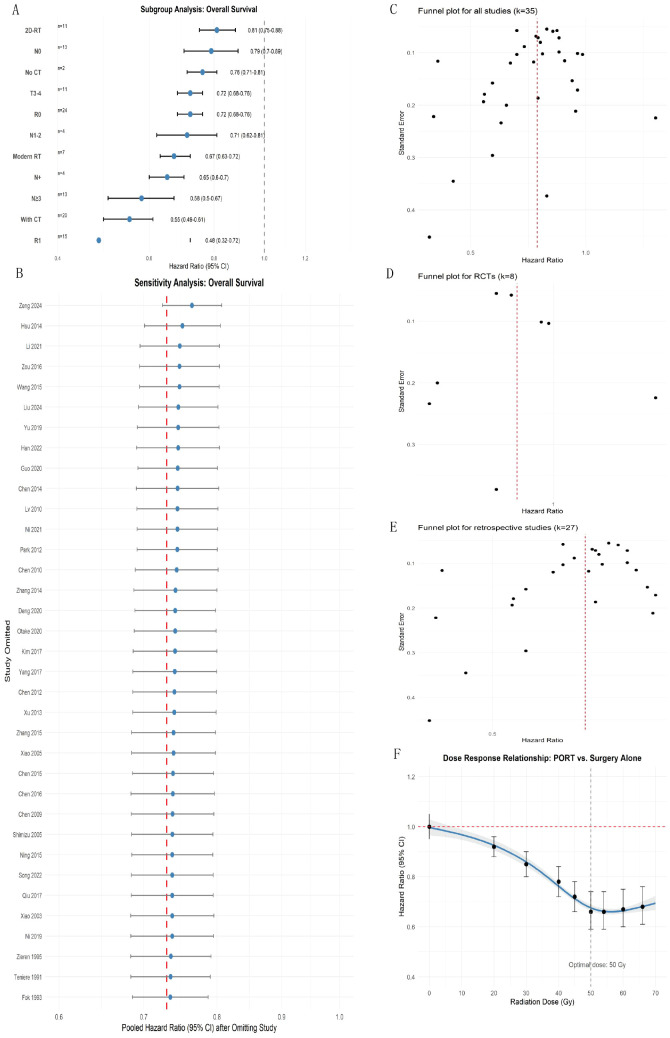
Subgroup, sensitivity, publication bias, and dose-response analyses. **(A)** Subgroup analysis for overall survival. **(B)** Sensitivity analysis of overall survival. **(C)** Funnel plot for all studies. **(D)** Funnel plot for RCTs. **(E)** Funnel plot for retrospective studies. **(F)** Dose-response relationship between radiation dose and survival.

Subgroup analyses demonstrated that PORT significantly improved OS in patients with positive lymph nodes (HR = 0.65), T3–4 stage disease (HR = 0.72), R0 resection (HR = 0.72), and those treated with modern radiotherapy (3D-CRT/IMRT, HR = 0.67). The greatest OS benefit was observed with PORT plus chemotherapy (HR = 0.55). Patients with ≥3 positive lymph nodes derived more benefit (HR = 0.58) than those with 1–2 positive nodes (HR = 0.71). Detailed subgroup results are shown in **Table 3**, **Figure 3A**.

**Table 3:** Subgroup analysis of overall survival.

Subgroup	Studies	HR (95% CI)	*I*² (%)	*P* value
Lymph node positive (N+)	13	0.65 (0.60–0.70)	35%	<0.001
Lymph node negative (N0)	7	0.79 (0.70–0.89)	30%	<0.001
T3–4 stage	13	0.72 (0.68–0.76)	0%	<0.001
Modern radiotherapy (3D-CRT/IMRT)	20	0.67 (0.63–0.72)	32%	<0.001
Conventional radiotherapy (2D-RT)	15	0.81 (0.75–0.88)	0%	<0.001
With chemotherapy	11	0.55 (0.49–0.61)	0%	<0.001
Without chemotherapy	24	0.76 (0.71–0.81)	39%	<0.001
≥3 positive nodes	4	0.58 (0.50–0.67)	0%	<0.001
1–2 positive nodes	4	0.71 (0.62–0.81)	0%	<0.001
R0 resection	11	0.72 (0.68–0.76)	0%	<0.001
R1 resection	2	0.48 (0.32–0.72)	0%	<0.001

#### Sensitivity analysis

To evaluate the robustness of our findings, we performed two sensitivity analyses. First, to assess the potential impact of HRs estimated from Kaplan-Meier curves, we excluded studies for which multivariable-adjusted HRs were not available (n = 14) (**Supplementary Table 5**). In this sensitivity analysis restricted to studies that directly reported multivariable-adjusted HRs (n=21), the pooled OS HR was 0.71 (95% CI: 0.67-0.76, *P* < 0.001, *I*²= 34%), which was similar to the main analysis result (HR = 0.74, 95% CI: 0.69-0.80, *P* < 0.001, *I*²= 67%). This confirms that the estimated HRs from Kaplan-Meier curves did not materially bias the pooled estimate, and the conclusion that PORT significantly improves OS remains robust. Second, we conducted a leave-one-out analysis by sequentially omitting each individual study; the pooled OS HRs ranged from 0.71 to 0.75, indicating that no single study disproportionately influenced the overall estimate (**Figure 3B**).

### Publication bias

Funnel plots were generated for all studies (**Figure 3C**), and separately for RCTs (**Figure 3D**) and retrospective studies (**Figure 3E**). Egger’s regression test was not significant for all studies (t = -1.46, df = 33, *P* = 0.154), for RCTs (t = 0.33, df = 6, *P* = 0.750), nor for retrospective studies (t = -1.96, df = 26, *P* = 0.061). These results indicate no evidence of substantial publication bias.

### Certainty of evidence (GRADE)

The certainty of evidence for the primary outcomes was evaluated using the GRADE framework. For overall survival, the evidence certainty was moderate, downgraded for serious inconsistency (*I*² = 67%). For disease−free survival, the certainty was moderate, downgraded for inconsistency (*I*² = 47%). For locoregional recurrence, the certainty was high (no inconsistency, direct evidence, no serious imprecision). A summary of findings table is provided in **Supplementary Table 6**.

### Meta-regression analysis

To explore potential sources of heterogeneity across studies, we performed meta-regression analyses incorporating the following covariates: use of chemotherapy (yes vs. no), publication year, study type (RCT vs. RS), and sample size. The results showed that the use of chemotherapy was a significant source of heterogeneity (coefficient = –0.208, 95% CI: –0.379 to –0.037, z = –2.39, *P* = 0.017), indicating that studies that included chemotherapy tended to report lower hazard ratios, suggesting a greater survival benefit from PORT. In contrast, publication year (coefficient = –0.003, 95% CI: –0.011 to 0.005, z = –0.75, *P* = 0.453), study type (coefficient = –0.052, 95% CI: –0.221 to 0.117, z = –0.60, *P* = 0.548), and sample size (coefficient = –0.0001, 95% CI: –0.0003 to 0.0001, z = –0.82, *P* = 0.412) did not significantly contribute to heterogeneity. To further explore potential sources of heterogeneity, we attempted to include additional covariates in the meta-regression analysis, including radiation dose (range 40-60 Gy), target volume definition (T-shaped field vs. tumor bed only), and surgical approach (two-field vs. three-field lymphadenectomy; left vs. right thoracotomy). However, these data were not consistently reported across the included studies, precluding a formal meta-regression. Consequently, we provide a qualitative discussion of their potential influence on heterogeneity in the Discussion section.

### Dose-response analysis

Dose-response analysis indicated a nonlinear relationship between radiation dose and survival benefit. Doses below 50 Gy were associated with insufficient local control, while doses above 50 Gy increased toxicity without additional survival gain. A dose of 50 Gy was identified as the optimal balance between efficacy and safety (**Figure 3F**), consistent with the findings of a study on adjuvant radiation dose in esophageal cancer (12).

## Discussion

To our knowledge, this is the largest updated meta-analysis (35 studies, 11,385 patients) confirming that postoperative radiotherapy (PORT) significantly improves overall survival (OS) and disease-free survival (DFS) and reduces locoregional recurrence (LRR) in patients with radically resected esophageal squamous cell carcinoma (ESCC), with the manageable acute toxicity. These findings are consistent with the results of the CROSS trial (41) and the NEOCRTEC5010 trial (42), which established the role of radiotherapy in multidisciplinary esophageal cancer management. Notably, the OS benefit of PORT in this study (HR = 0.74) was comparable to that of neoadjuvant chemoradiotherapy in the CROSS trial (HR = 0.67) (43), suggesting that PORT is a valuable adjuvant option for patients who undergo upfront surgery. Moreover, several other phase III trials comparing neoadjuvant chemoradiotherapy with surgery alone have consistently demonstrated survival benefits of neoadjuvant therapy (44–47). The comparable efficacy between PORT and neoadjuvant regimens further supports the role of radiotherapy as a key component of esophageal cancer treatment, whether given before or after surgery.

PORT reduced the risk of death by 26%, disease progression by 38%, and locoregional recurrence by 70%, with enhanced benefits in patients treated with modern radiotherapy (3D-CRT/IMRT) or combined chemotherapy. Although local control was significantly improved, there was no reduction in distant metastasis (OR = 1.01, *P* = 0.85), consistent with the classic RCT that showed that PORT significantly reduced intrathoracic recurrence (from 25.9% to 16.2%, *P* = 0.015) and supraclavicular recurrence (from 13.2% to 3.1%, *P* = 0.001) but not distant metastasis (5). This suggests that PORT acts primarily at the local-regional level and has limited ability to control systemic micrometastases; therefore, adding systemic chemotherapy is critical for high-risk patients.

This conclusion is supported by multiple high-quality studies. A phase III RCT (172 patients) showed that PORT combined with chemotherapy significantly improved DFS (HR = 0.59) and OS (HR = 0.60) in stage IIB-III patients (13). Another study using IMRT (506 patients) reported a 5-year OS of 53.8% in the PORT group compared with 25.3% in the surgery-alone group (HR = 0.41), the largest survival benefit reported to date (14), similar to the 5-year OS of 47% with neoadjuvant chemoradiotherapy in CROSS (43). A propensity-matched study (290 patients) showed that postoperative chemoradiotherapy improved 3-year OS from 16.8% to 48.6% (HR = 0.40) (37). The newly included studies further reinforced these findings: a phase III RCT showed that PORT significantly improved DFS in pT2-3N0M0 patients (HR = 0.53) (15); a large PSM study showed a 36.5% reduction in mortality (HR = 0.635) (16); and another study confirmed a significant benefit in pN+ patients (HR = 0.543) (17). Collectively, these results indicate that with modern radiotherapy techniques, PORT can achieve survival benefits comparable to neoadjuvant chemoradiotherapy.

Safety analysis showed that PORT increased the risk of grade ≥3 acute toxicity (OR = 2.45, *P* < 0.001), primarily radiation esophagitis (12.5%) and leukopenia (15.2%). However, severe (grade ≥4) toxicity was rare (<5%), and treatment-related deaths were very low (0.5%) and not significantly different from those in surgery-alone group. This contrasts sharply with early studies (6) that reported gastrointestinal complication rates as high as 37% and several treatment-related deaths, highlighting the tremendous improvement in safety achieved by modern radiotherapy techniques. The adoption of intensity-modulated radiotherapy (IMRT) and three-dimensional conformal radiotherapy (3D-CRT) has been shown to significantly reduce radiation exposure to organs at risk (48, 49). A systematic review further confirmed that IMRT provides superior target coverage and lower normal tissue doses compared with 3D-CRT (50). These dosimetric advantages translate into improved treatment tolerance and quality of life for patients.

Subgroup analyses identified the populations that derive the greatest benefit. Patients with positive lymph nodes derived the most significant OS benefit from PORT (HR = 0.65), while those with negative nodes had limited benefit (HR = 0.79). Further stratification showed that patients with ≥3 positive nodes had the most significant benefit (HR = 0.58), consistent with the classic study that showed PORT improved 5-year OS from 0% to 20.6% in patients with ≥3 positive nodes (19). Patients with T3–4 stage disease also derived significant benefit (HR = 0.72) (10), and those with R0 resection (HR = 0.72) (25). Patients with R1 resection also benefited from PORT (22), though combined chemoradiotherapy is preferred to compensate for positive margins. These results support risk-adapted adjuvant strategies.

The moderate-to-high heterogeneity observed for overall survival (*I*² = 67%) may be partly explained by several clinical factors that could not be quantitatively explored due to inconsistent reporting. The prescribed dose ranged from 40 to 60 Gy across studies. Although our dose-response analysis suggested 50 Gy as the optimal balance between efficacy and toxicity, dose variations could influence local control and survival, potentially contributing to heterogeneity. Some studies used large T-shaped fields covering bilateral supraclavicular and entire mediastinal regions, while others used smaller fields limited to the tumor bed and adjacent nodes. Larger target volumes may improve locoregional control but also increase toxicity, affecting the consistency of effect estimates. Differences in lymphadenectomy extent (Two-field vs. Three-field) and thoracotomy side (left vs. right) may alter the completeness of resection and the pattern of residual disease, thereby modifying the benefit of PORT. Moreover, differences in chemotherapy regimens (e.g., cisplatin-fluorouracil vs. Paclitaxel-cisplatin) and patient baseline characteristics (age, performance status, tumor length) may also have contributed to the observed heterogeneity.

Notably, in the subgroup receiving modern radiotherapy (IMRT/3D-CRT), heterogeneity was moderate (*I*² = 32%), whereas in the conventional 2D-RT subgroup, heterogeneity was absent (*I*² = 0%). This difference may reflect that older 2D-RT studies were more uniform in patient selection, target volume delineation, and treatment protocols, while modern techniques are applied across a wider range of clinical scenarios (e.g., various dose prescriptions, different target volume definitions, and combined chemotherapy regimens), leading to greater variability in outcomes. This observation underscores that the overall heterogeneity in the primary analysis (*I*² = 67%) is partly driven by heterogeneity within the modern radiotherapy subgroup.

Previous studies have shown that postoperative recurrences predominantly occur in the supraclavicular and upper mediastinal regions, supporting the use of T-shaped target volumes for PORT (51–56). Our meta-analysis confirms that PORT significantly reduces locoregional recurrence (OR = 0.30, 95% CI: 0.27-0.34, *P* < 0.001). This finding aligns with the anatomical rationale that PORT effectively covers these high-risk areas, thereby improving local control. Clinically, these results advocate for the routine inclusion of bilateral supraclavicular and upper mediastinal regions in PORT target volume design, especially for patients with upper or middle thoracic ESCC.

The optimal timing of PORT remains debated. Some evidence suggests that delaying PORT beyond 48 days or using sequential chemoradiotherapy may be beneficial (57, 58); however, these observations are based on external cohorts and were not directly evaluated in our meta-analysis. Our study demonstrates that PORT, when administered postoperatively, significantly improves OS and DFS regardless of timing variations across studies. Future prospective trials are needed to determine the optimal sequence and timing of PORT.

Modern radiotherapy techniques (IMRT/3D-CRT) were associated with a superior OS benefit (HR = 0.67) compared with conventional 2D-RT (HR = 0.81), and with lower toxicity. One study using IMRT achieved the largest survival benefit reported to date (HR = 0.41) (14), whereas an earlier trial using 2D-RT even showed harm from PORT (HR = 1.52) (6). The choice of target volume also influences outcomes. A study comparing regional (tumor bed and adjacent nodes) versus extensive (including supraclavicular, entire mediastinum, anastomosis, and left gastric/pericardial areas) PORT found no significant survival difference, but the extensive field was associated with higher toxicity (59). A randomized phase II trial demonstrated that elective nodal irradiation (including the supraclavicular and upper mediastinal regions) significantly improved local control without increasing severe toxicity compared with tumor bed-only irradiation (60). Another phase II trial further confirmed the feasibility and safety of an extensive clinical target volume (61). These studies indicate that while modern techniques allow for larger target volumes with acceptable toxicity, careful individualization is still necessary. Dose-response analysis confirmed a nonlinear relationship between radiation dose and survival benefit, with 50 Gy identified as the optimal balance between local control and toxicity. This is consistent with the landmark RTOG 94-05 trial, which compared high-dose (64.8 Gy) versus standard-dose (50.4 Gy) chemoradiotherapy for inoperable esophageal cancer and found no survival benefit with dose escalation but significantly higher toxicity (62). Therefore, the current evidence supports 50 Gy as the standard dose for PORT.

Combined chemotherapy provided the greatest OS benefit (HR = 0.55), far superior to PORT alone (HR = 0.76). This advantage is supported by multiple studies. One study reported that postoperative chemoradiotherapy significantly improved 5-year OS compared with PORT alone (38.0% vs. 29.6%, *P* = 0.001) (63). Another study showed that the distant metastasis rate was significantly lower in the POCRT group (18.9%) than in the PORT-alone group (26.4%, *P* = 0.03) (34). A further study also confirmed that adjuvant chemoradiotherapy provided better survival outcomes than radiotherapy alone after R0 resection (40).

The benefit can be explained by two mechanisms: first, chemotherapy enhances radiosensitivity through cell cycle synchronization and inhibition of DNA repair; second, chemotherapy eradicates systemic micro metastases, compensating for the limited ability of PORT to control distant diseases. For patients with good performance status, postoperative chemoradiotherapy may be considered a reasonable adjuvant approach (18, 31).

This study has several limitations. Most included studies were retrospective, which may introduce selection bias. Although we performed subgroup analyses and meta-regression to explore heterogeneity, unmeasured confounders may remain. The number of high-quality RCTs remains limited, and some older trials used outdated radiotherapy techniques, which may not reflect current practice. Variations in radiotherapy dose, target volume, and chemotherapy regimens across studies may contribute to heterogeneity. Some HRs were estimated from Kaplan-Meier curves, potentially introducing error. Safety data reporting was not uniform across studies; some did not provide detailed toxicity grading. Moreover, late toxicities (e.g., radiation-induced pulmonary fibrosis, pericardial effusion) were rarely reported in the included studies, precluding a meta-analysis; future prospective studies should systematically collect late toxicity data. Biomarker studies are still ongoing and require further validation. Finally, direct comparisons between PORT and neoadjuvant therapy are scarce, so conclusions regarding comparative effectiveness should be interpreted with caution.

## Conclusions

PORT significantly improves OS and DFS and reduces locoregional recurrence in radically resected ESCC, particularly in high-risk patients with lymph node positivity, T3–4 stage disease, or R0 resection. Modern radiotherapy (3D-CRT/IMRT) at a dose of 50 Gy is recommended. Although PORT increases acute toxicity, such events are manageable. PORT represents an effective and safe adjuvant strategy for appropriately selected patients.

## Data Availability

The original contributions presented in the study are included in the article/**Supplementary Material**. Further inquiries can be directed to the corresponding authors.

## Supplementary material

Supplementary Figure 1 ROB2 traffic-light plot and weighted bar plot.

Supplementary Table 1 Complete search strategies for each database.

Supplementary Table 2 .

Supplementary Table 3 Jadad Scale Scores for Randomized Controlled Trials (8 Studies). * Randomization (0–2 points): 1 point if described, +1 if appropriate, –1 if inappropriate. Blinding (0–2 points): 1 point if described, +1 if appropriate. Withdrawals & Dropouts (0–1 point): 1 point if described. Studies with total ≥4 are considered high quality.*.

Supplementary Table 4 Newcastle-Ottawa Scale (NOS) Scores for Retrospective Studies (27 Studies). Selection (0–4 stars), Comparability (0–2 stars), Outcome (0–3 stars). Studies with total ≥6 are considered high quality.

Supplementary Table 5 Studies excluded from sensitivity analysis for HR estimation method (n=14).

Supplementary Table 6 GRADE summary of findings.

Supplementary Table 7 Excluded full-text articles (N=136).
