# Supplementary figures and images for: Postoperative radiotherapy for resected esophageal squamous cell carcinoma: a systematic review and meta-analysis
Source: Front Oncol. 2026 Jul 14;16:1878657. doi: 10.3389/fonc.2026.1878657 (PMC13407294; doi:10.3389/fonc.2026.1878657)

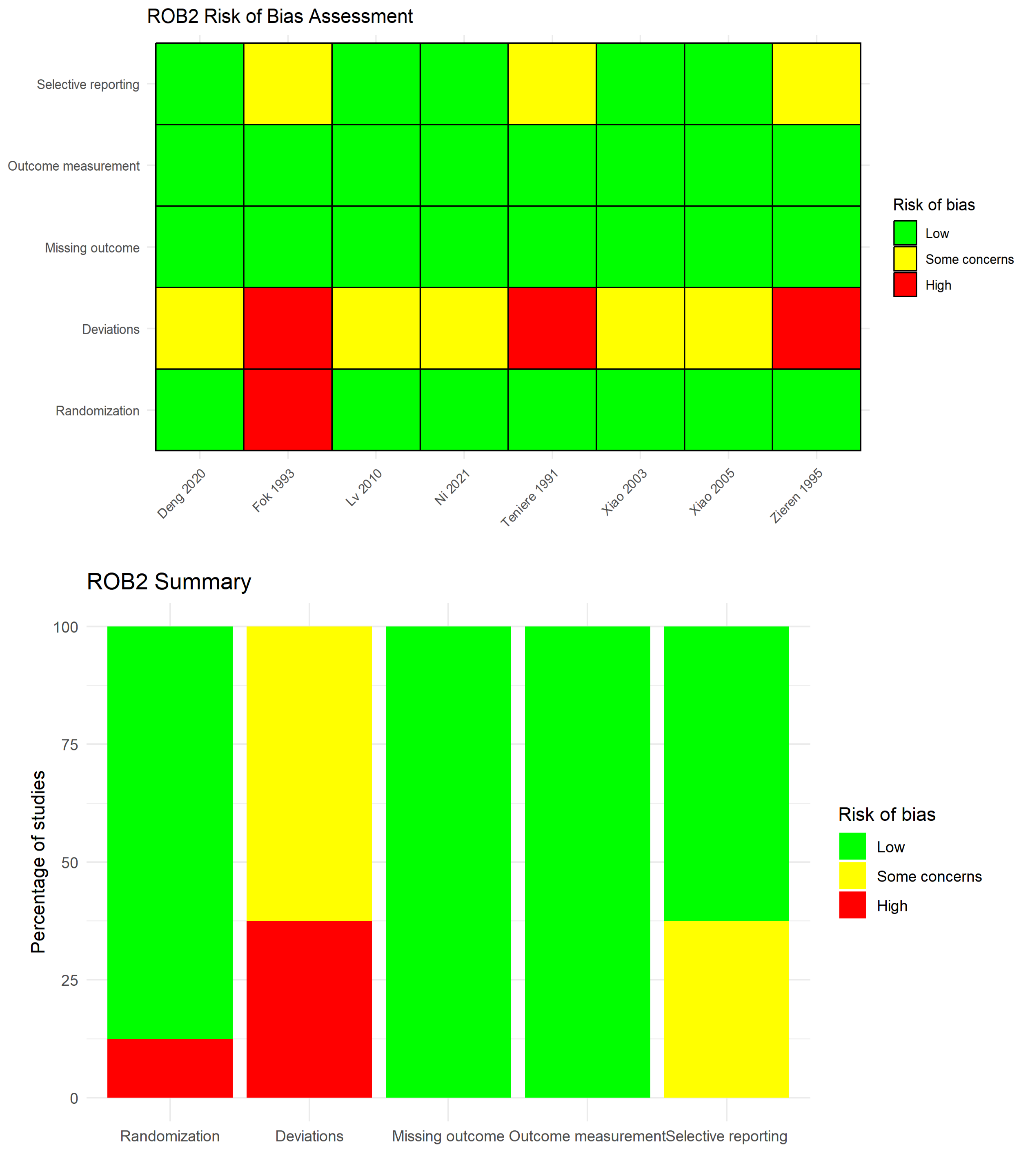

Supplement: Supplementary Figure 1 — ROB2 traffic-light plot and weighted bar plot. [file Image1.tif]
