# Supplementary material for: Postoperative radiotherapy for resected esophageal squamous cell carcinoma: a systematic review and meta-analysis
Source: Front Oncol. 2026 Jul 14;16:1878657. doi: 10.3389/fonc.2026.1878657 (PMC13407294; doi:10.3389/fonc.2026.1878657)
Supplement: Supplementary Table 1 — Complete search strategies for each database. [file Table1.docx]

**Supplementary Table S1. Complete search strategies for each database**

| Database | Search date | Search strategy |
| --- | --- | --- |
| PubMed | 1990/01/01 – 2025/06/30 | ("Esophageal Neoplasms"[Mesh] OR "Esophageal Squamous Cell Carcinoma"[Mesh] OR "esophagus cancer"[tiab] OR "esophageal cancer"[tiab] OR "ESCC"[tiab]) AND ("Radiotherapy"[Mesh] OR "Radiotherapy, Adjuvant"[Mesh] OR "postoperative radiotherapy"[tiab] OR "adjuvant radiotherapy"[tiab] OR "PORT"[tiab]) AND ("Surgery"[Mesh] OR "Esophagectomy"[Mesh] OR "surgery alone"[tiab] OR "esophagectomy alone"[tiab]) |
| EMBASE | 1990/01/01 – 2025/06/30 | ('esophageal cancer'/exp OR 'esophagus cancer':ti,ab OR 'esophageal squamous cell carcinoma':ti,ab OR 'escc':ti,ab) AND ('radiotherapy'/exp OR 'adjuvant radiotherapy':ti,ab OR 'postoperative radiotherapy':ti,ab OR 'port':ti,ab) AND ('surgery'/exp OR 'esophagectomy'/exp OR 'surgery alone':ti,ab OR 'esophagectomy alone':ti,ab) |
| Cochrane Library | 1990/01/01 – 2025/06/30 | (MeSH descriptor: [Esophageal Neoplasms] OR MeSH descriptor: [Esophageal Squamous Cell Carcinoma] OR (esophagus cancer):ti,ab OR (esophageal cancer):ti,ab OR (ESCC):ti,ab) AND (MeSH descriptor: [Radiotherapy] OR MeSH descriptor: [Radiotherapy, Adjuvant] OR (postoperative radiotherapy):ti,ab OR (adjuvant radiotherapy):ti,ab OR (PORT):ti,ab) AND (MeSH descriptor: [Surgery] OR MeSH descriptor: [Esophagectomy] OR (surgery alone):ti,ab OR (esophagectomy alone):ti,ab) |

### Supplementary Table S2

| Study | Domain 1 (Randomization) | Domain 2 (Deviations) | Domain 3 (Missing data) | Domain 4 (Outcome measurement) | Domain 5 (Selective reporting) | Overall |
| --- | --- | --- | --- | --- | --- | --- |
| Xiao 2003 | Low | Some concerns | Low | Low | Low | Some concerns |
| Xiao 2005 | Low | Some concerns | Low | Low | Low | Some concerns |
| Zieren 1995 | Low | High | Low | Low | Some concerns | High |
| Teniere 1991 | Low | High | Low | Low | Some concerns | High |
| Lv 2010 | Low | Some concerns | Low | Low | Low | Some concerns |
| Ni 2021 | Low | Some concerns | Low | Low | Low | Some concerns |
| Deng 2020 | Low | Some concerns | Low | Low | Low | Some concerns |
| Fok 1993 | High | High | Low | Low | Some concerns | High |

### Supplementary Table S3. Jadad Scale Scores for Randomized Controlled Trials (8 Studies)

| Study | Randomization | Randomization Appropriate | Blinding | Blinding Appropriate | Withdrawals & Dropouts | **Total (0–7)** | **Quality** |
| --- | --- | --- | --- | --- | --- | --- | --- |
| Xiao 2003 | 1 | 1 | 0 | 0 | 1 | **5** | High |
| Xiao 2005 | 1 | 1 | 0 | 0 | 1 | **5** | High |
| Zieren 1995 | 1 | 1 | 0 | 0 | 1 | **4** | High |
| Teniere 1991 | 1 | 1 | 0 | 0 | 1 | **4** | High |
| Lv 2010 | 1 | 1 | 0 | 0 | 1 | **5** | High |
| Ni 2021 | 1 | 1 | 0 | 0 | 1 | **6** | High |
| Deng 2020 | 1 | 1 | 0 | 0 | 1 | **6** | High |
| Fok 1993 | 1 | -1 (inappropriate) | 0 | 0 | 1 | **3** | Low |

*Note: Randomization (0–2 points): 1 point if described, +1 if appropriate, –1 if inappropriate. Blinding (0–2 points): 1 point if described, +1 if appropriate. Withdrawals & Dropouts (0–1 point): 1 point if described. Studies with total ≥4 are considered high quality.*

### Supplementary Table S4. Newcastle-Ottawa Scale (NOS) Scores for Retrospective Studies (27 Studies)

| First Author | Year | Selection (0–4) | Comparability (0–2) | Outcome (0–3) | **Total (0–9)** |
| --- | --- | --- | --- | --- | --- |
| Chen | 2016 | ★★★ | ★★ | ★★ | **7** |
| Yang | 2017 | ★★★ | ★★ | ★★★ | **8** |
| Ni | 2019 | ★★★ | ★★ | ★★ | **7** |
| Qiu | 2017 | ★★★ | ★ | ★★ | **6** |
| Zhang | 2015 | ★★★ | ★★ | ★★★ | **8** |
| Kim | 2017 | ★★★ | ★★ | ★★★ | **8** |
| Chen | 2014 | ★★★ | ★★ | ★★ | **7** |
| Chen | 2009 | ★★★ | ★ | ★★ | **6** |
| Chen | 2012 | ★★★ | ★★ | ★★ | **7** |
| Shimizu | 2005 | ★★ | ★★ | ★★ | **6** |
| Han | 2022 | ★★★★ | ★★ | ★★ | **8** |
| Otake | 2020 | ★★★ | ★★ | ★★ | **7** |
| Zhang | 2014 | ★★★ | ★★ | ★★ | **7** |
| Song | 2022 | ★★★ | ★★ | ★★ | **7** |
| Guo | 2020 | ★★★ | ★★ | ★★ | **7** |
| Wang | 2015 | ★★★ | ★★ | ★★ | **7** |
| Liu | 2024 | ★★★ | ★★ | ★★ | **7** |
| Chen | 2010 | ★★★ | ★★ | ★★ | **7** |
| Zou | 2016 | ★★★ | ★★ | ★★ | **7** |
| Chen | 2015 | ★★★ | ★★ | ★★ | **7** |
| Xu | 2013 | ★★★ | ★★ | ★★ | **7** |
| Zeng | 2024 | ★★★★ | ★★ | ★★ | **8** |
| Hsu | 2014 | ★★★ | ★★ | ★★ | **7** |
| Park | 2012 | ★★★ | ★ | ★★ | **6** |
| Ning | 2015 | ★★★ | ★★ | ★★ | **7** |
| Yu | 2019 | ★★★★ | ★★ | ★★ | **8** |
| Li | 2021 | ★★★★ | ★★ | ★★ | **8** |

Note: Selection (0–4 stars), Comparability (0–2 stars), Outcome (0–3 stars). Studies with total ≥6 are considered high quality.

## Supplementary Table S5. Studies excluded from sensitivity analysis for HR estimation method (n=14)

| No. | First author | Year | Reason for exclusion |
| --- | --- | --- | --- |
| 1 | Zieren HU | 1995 | RCT; no multivariable-adjusted HR; HR estimated from KM curve |
| 2 | Teniere P | 1991 | RCT; no multivariable-adjusted HR; HR estimated from KM curve |
| 3 | Qiu B | 2017 | Retrospective; no multivariable-adjusted HR; HR estimated from KM curve |
| 4 | Shimizu K | 2005 | Retrospective; small sample; no multivariable-adjusted HR; HR estimated from KM curve |
| 5 | Chen G | 2009 | Retrospective; no multivariable-adjusted HR; HR estimated from KM curve |
| 6 | Guo Y | 2020 | Retrospective; wide CI; no multivariable-adjusted HR; HR estimated from KM curve |
| 7 | Chen X | 2015 | Retrospective; no multivariable-adjusted HR; HR estimated from KM curve |
| 8 | Park IJ | 2012 | Retrospective; small sample; no multivariable-adjusted HR; HR estimated from KM curve |
| 9 | Ning ZH | 2015 | Retrospective; subgroup HR estimated from KM curve; no multivariable HR for subgroup |
| 10 | Zhang W | 2015 | Retrospective; subgroup HR estimated from KM curve; no multivariable HR for subgroup |
| 11 | Otake R | 2020 | Retrospective; OS HR estimated from KM curve (LPSS/PSS had multivariable but not OS) |
| 12 | Lv J | 2010 | RCT; no multivariable-adjusted HR; HR estimated from KM curve |
| 13 | Chen SB | 2016 | Retrospective; OS HR estimated from KM curve (no multivariable adjustment reported) |
| 14 | Song C | 2022 | Retrospective (PSM); no multivariable-adjusted HR; HR estimated from KM curve |

**Supplementary Table S6. GRADE summary of findings**

| Outcome | No. of studies | Effect estimate (95% CI) | Certainty | Reason for downgrading |
| --- | --- | --- | --- | --- |
| OS | 35 | HR 0.74 (0.69‑0.80) | Moderate | Inconsistency (*I*²=67%) |
| DFS | 22 | HR 0.62 (0.58‑0.67) | Moderate | Inconsistency (*I*²=47%) |
| LRR | 24 | OR 0.30 (0.27‑0.34) | High | none |

### Supplementary Table S7. Excluded full-text articles (N=136)

| No. | First author | Year | Title | Exclusion reason |
| --- | --- | --- | --- | --- |
| 1 | Ai D | 2023 | Extensive clinical target volume in postoperative chemoradiotherapy for esophageal squamous cell carcinoma: a phase II clinical trial (ESO-Shanghai 9) | No OS/DFS data |
| 2 | Cai WJ | 2010 | Pattern of relapse in surgical treated patients with thoracic esophageal squamous cell carcinoma and its possible impact on target delineation for postoperative radiotherapy | No OS/DFS data |
| 3 | Cai XW | 2019 | Randomized phase II trial comparing tumor bed alone with tumor bed and elective nodal postoperative radiotherapy in patients with locoregionally advanced thoracic esophageal squamous cell carcinoma | No OS/DFS data |
| 4 | Chang X | 2022 | Comparison of Two Major Staging Systems in Predicting Survival and Recommendation of Postoperative Radiotherapy Based on the 11th Japanese Classification for Esophageal Carcinoma After Curative Resection: A Propensity Score-Matched Analysis | HR not available |
| 5 | Chen H | 2013 | Prospective study of adjuvant radiotherapy on preventing lymph node metastasis after Ivor-Lewis esophagectomy in esophageal cancer | No OS/DFS data |
| 6 | Chen J | 2013 | Postoperative radiation therapy with or without concurrent chemotherapy for node-positive thoracic esophageal squamous cell carcinoma | No OS/DFS data |
| 7 | Chen J | 2014 | Prognostic analysis of cervical lymph node metastasis in patients with thoracic esophageal squamous cell carcinoma | Overlapping population |
| 8 | Chen J | 2017 | Patterns and rates of abdominal lymphatic metastasis following esophageal carcinoma | No OS/DFS data |
| 9 | Chen Z | 2020 | Identification of critical radioresistance genes in esophageal squamous cell carcinoma by whole-exome sequencing | No OS/DFS data |
| 10 | Cui T | 2021 | Pattern of Recurrence in 428 Patients With Thoracic Esophageal Squamous Cell Carcinoma After Radical Surgery and Its Implication in Postoperative Radiotherapeutic Clinical Target Volume | No OS/DFS data |
| 11 | Dai D | 2022 | PELI1 promotes radiotherapy sensitivity by inhibiting noncanonical NF-κB in esophageal squamous cancer | No OS/DFS data |
| 12 | Ding W | 2020 | Postoperative radiotherapy for the young-old patients with thoracic esophageal squamous cell carcinoma: A 2-center experience | No OS/DFS data |
| 13 | Elias D | 1983 | Cancer of the cervical esophagus. 64 cases treated by radiotherapy. Evaluation and considerations | No English |
| 14 | Fu JH | 2009 | Attaching importance to clinical study of neo-adjuvant chemoradiotherapy followed by surgery for esophageal squamous cell carcinoma | No English |
| 15 | Gao HM | 2022 | Construction of a predictive model of abdominal lymph node metastasis in thoracic esophageal squamous cell carcinoma and preliminary analysis of its effect on target for postoperative radiotherapy | No OS/DFS data |
| 16 | Gao M | 2022 | Effect of different surgical approaches on the prognosis of patients with postoperative radiotherapy for stage IIB-IVA esophageal squamous cancer | No OS/DFS data |
| 17 | Guo JC | 2024 | Adjuvant chemoradiotherapy plus pembrolizumab for locally advanced esophageal squamous cell carcinoma with high risk of recurrence following neoadjuvant chemoradiotherapy: a single-arm phase II study | No OS/DFS data |
| 18 | He W | 2025 | Long-Term Survival Outcomes of NCRT With Surgery vs Surgery With Adjuvant Therapy for ESCC: A Single-Center Prospective Phase 3 Randomized Clinical Trial | No OS/DFS data |
| 19 | Hilgenberg AD | 1988 | Preoperative chemotherapy, surgical resection, and selective postoperative therapy for squamous cell carcinoma of the esophagus | No OS/DFS data |
| 20 | Hsu FM | 2008 | Improved local control by surgery and paclitaxel-based chemoradiation for esophageal squamous cell carcinoma: results of a retrospective non-randomized study | No OS/DFS data |
| 21 | Huang PM | 2017 | Do We Need to Add Postoperative Radiotherapy in Patients Undergoing Trimodality Therapy for Esophageal Squamous Cell Carcinoma with Positive Lymph Nodes Disease? | No OS/DFS data |
| 22 | Huang S | 2019 | Investigating the Survival Benefit of Combining Radiotherapy for Surgery Treated Locally Advanced Esophageal Squamous Cell Carcinoma Patients Aged 65 and Older | No OS/DFS data |
| 23 | Hwang JY | 2015 | A Propensity-matched Analysis Comparing Survival After Esophagectomy Followed by Adjuvant Chemoradiation to Surgery Alone for Esophageal Squamous Cell Carcinoma | Overlapping population |
| 24 | Ikeguchi M | 2001 | Bax expression as a prognostic marker of postoperative chemoradiotherapy for patients with esophageal cancer | No OS/DFS data |
| 25 | Jiang C | 2019 | High PD-L1 expression is associated with a favorable prognosis in patients with esophageal squamous cell carcinoma undergoing postoperative adjuvant radiotherapy | HR not available |
| 26 | Jiang W | 2019 | Evaluation of surgery plus postoperative radiotherapy or definitive radiotherapy in older patients with thoracic esophageal squamous cell cancer | No OS/DFS data |
| 27 | Kang M | 2025 | Patterns of Lymph Node Recurrence after Esophagectomy of pT2-3 N0M0 Esophageal Squamous Cell Carcinoma | No OS/DFS data |
| 28 | Koterazawa Y | 2025 | A comparison of the clinical outcomes of esophagectomy and chemoradiotherapy for patients aged 75 years or older with surgically resectable esophageal squamous cell carcinoma | No OS/DFS data |
| 29 | Koyanagi K | 2021 | Progress in Multimodal Treatment for Advanced Esophageal Squamous Cell Carcinoma: Results of Multi-Institutional Trials Conducted in Japan | No OS/DFS data |
| 30 | Laufer I | 2013 | Local disease control for spinal metastases following "separation surgery" and adjuvant hypofractionated or high-dose single-fraction stereotactic radiosurgery | No OS/DFS data |
| 31 | Lertbutsayanukul C | 2017 | High dose radiation with chemotherapy followed by salvage esophagectomy among patients with locally advanced esophageal squamous cell carcinoma | No OS/DFS data |
| 32 | Li C | 2021 | Clinical practice and outcome of radiotherapy for advanced esophageal squamous cell carcinoma between 2002 and 2018 in China: the multi-center 3JECROG Survey | No OS/DFS data |
| 33 | Li H | 2022 | Postoperative Concurrent Chemoradiotherapy for Locally Advanced Thoracic Esophageal Squamous Cell Carcinoma: A Phase II Clinical Trial | No OS/DFS data |
| 34 | Li L | 2017 | Adjuvant Therapeutic Modalities Following Three-field Lymph Node Dissection for Stage II/III Esophageal Squamous Cell Carcinoma | No OS/DFS data |
| 35 | Li X | 2023 | Cytoplasmic poly(A)-binding protein 1 as a biomarker to assist early diagnosis and prognosis of esophageal squamous cell carcinoma in endoscopic biopsy fragments | No OS/DFS data |
| 36 | Li Y | 2019 | Predicting the Value of Adjuvant Therapy in Esophageal Squamous Cell Carcinoma by Combining the Total Number of Examined Lymph Nodes with the Positive Lymph Node Ratio | No OS/DFS data |
| 37 | Liang YH | 2023 | Lymph node ratio precisely predicts the benefit of postoperative radiotherapy in esophageal cancer: A retrospective cohort study | HR not available |
| 38 | Lin MQ | 2021 | Delayed postoperative radiotherapy might improve the long-term prognosis of locally advanced esophageal squamous cell carcinoma | No OS/DFS data |
| 39 | Lin Y | 2014 | Value of postoperative radiochemotherapy for thoracic esophageal squamous cell carcinoma with lymph node metastasis | No English |
| 40 | Ling Y | 2012 | Combined influence of adjuvant therapy and interval after surgery on peripheral CD4(+) T lymphocytes in patients with esophageal squamous cell carcinoma | No OS/DFS data |
| 41 | Liu D | 2025 | Postoperative Adjuvant Therapy Benefits Non-pCR Patients Rather Than pCR Patients for Locally Advanced ESCC: A Multicenter Real-World Study | No OS/DFS data |
| 42 | Liu J | 2017 | Characteristics of the local recurrence pattern after curative resection and values in target region delineation in postoperative radiotherapy for lower thoracic esophageal squamous cell cancer | No OS/DFS data |
| 43 | Liu Q | 2014 | Patterns of failure after radical surgery among patients with thoracic esophageal squamous cell carcinoma: implications for the clinical target volume design of postoperative radiotherapy | No OS/DFS data |
| 44 | Liu R | 2021 | Adjuvant Radiotherapy of Involved Field versus Elective Lymph Node in Patients with Operable Esophageal Squamous Cell Cancer: A Single Institution Prospective Randomized Controlled Study | No OS/DFS data |
| 45 | Liu T | 2018 | The role of postoperative radiotherapy for radically resected esophageal squamous cell carcinoma: a systemic review and meta-analysis | No OS/DFS data |
| 46 | Liu W | 2025 | Salvage involved-field versus extended-field chemoradiotherapy for postoperative lymph node metastasis in esophageal squamous cell carcinoma | No OS/DFS data |
| 47 | Lu H | 2024 | A retrospective cohort study to observe the efficacy and safety of Endoscopic Submucosal Dissection (ESD) with adjuvant radiotherapy for T1a-MM/T1b-SM Esophageal Squamous Cell Carcinoma | No OS/DFS data |
| 48 | Lu JC | 2008 | Extent of prophylactic postoperative radiotherapy after radical surgery of thoracic esophageal squamous cell carcinoma | No OS/DFS data |
| 49 | Lu JC | 2009 | Prognostic factors of radiotherapy in patients with node-positive thoracic esophageal squamous cell carcinoma after radical surgery | No OS/DFS data |
| 50 | Lu JC | 2010 | Radiotherapy with or without concurrent chemotherapy for lymph node recurrence after radical surgery of thoracic esophageal squamous cell carcinoma | No OS/DFS data |
| 51 | Luo Y | 2016 | Postoperative radiation therapy of pT2-3N0M0 esophageal carcinoma-a review | No OS/DFS data |
| 52 | Lyu X | 2014 | Adjuvant Chemotherapy After Esophagectomy: Is There a Role in the Treatment of the Lymph Node Positive Thoracic Esophageal Squamous Cell Carcinoma? | HR not available |
| 53 | Mayanagi S | 2024 | Survival outcome of esophagectomy and chemoradiotherapy for resectable esophageal squamous cell carcinoma in patients >75 years of age | No OS/DFS data |
| 54 | Mu Y | 2025 | Efficacy and safety of postoperative radiotherapy in locally advanced esophageal squamous cell carcinoma patients with pathologic incomplete response after neoadjuvant immunochemotherapy | No OS/DFS data |
| 55 | Murakami M | 2000 | Comparison between chemoradiation protocol intended for organ preservation and conventional surgery for clinical T1-T2 esophageal carcinoma | No OS/DFS data |
| 56 | Ni W | 2020 | A phase-II/III randomized controlled trial of adjuvant radiotherapy or concurrent chemoradiotherapy after surgery versus surgery alone in patients with stage-IIB/III esophageal squamous cell carcinoma (protocol) | No OS/DFS data |
| 57 | Ning ZH | 2015 | The status of perineural invasion predicts the outcomes of postoperative radiotherapy in locally advanced esophageal squamous cell carcinoma | HR not available |
| 58 | Ohira M | 2015 | Impact of Chemoradiation-induced Myelosuppression on Prognosis of Patients with Locally Advanced Esophageal Cancer After Chemoradiotherapy Followed by Esophagectomy | No OS/DFS data |
| 59 | Pan WB | 2017 | Associated factors of postoperative relapse and metastasis in pT1bN0M0-pT4aN0M0 thoracic esophageal squamous cell carcinoma | No English |
| 60 | Qiao XY | 2008 | Comparison of efficacy of regional and extensive clinical target volumes in postoperative radiotherapy for esophageal squamous cell carcinoma | No OS/DFS data |
| 61 | Sakin A | 2021 | Chemoradiotherapy followed by surgery versus observation in esophageal squamous cell carcinoma | No OS/DFS data |
| 62 | Shao GG | 2016 | Efficacy of adjuvant therapy in 110 patients with N1 lymph node metastasis of esophageal squamous cell carcinoma | No English |
| 63 | Shen JY | 2025 | Postoperative chemoradiotherapy for esophageal squamous cell Carcinoma: Results from ESO-Shanghai 17 and joint analyses for phase II clinical trials | No OS/DFS data |
| 64 | Shen WB | 2017 | Analysis of the causes of failure after radical surgery in patients with PT3N0M0 thoracic esophageal squamous cell carcinoma and consideration of postoperative radiotherapy | No OS/DFS data |
| 65 | Shen WB | 2019 | Dosimetric Predictors of Radiation Gastritis Due to Postoperative Intensity Modulated Irradiation Therapy in Patients with Esophageal Squamous Cell Carcinoma After Radical Esophagectomy | No OS/DFS data |
| 66 | Shueng PW | 2022 | Combined modality therapy for patients with esophageal squamous cell carcinoma: Radiation dose and survival analyses | No OS/DFS data |
| 67 | Song T | 2020 | The Role of Adjuvant Chemoradiotherapy Over Radiotherapy After R0 Resection for Stage II-III Esophageal Squamous Cell Carcinoma | No OS/DFS data |
| 68 | Song XY | 2025 | Radiotherapy as an organ-preserving alternative to surgery in patients with locally advanced esophageal squamous cell carcinoma achieving major pathologic response after induction immunochemotherapy | No OS/DFS data |
| 69 | Tachimori Y | 2009 | Salvage esophagectomy after high-dose chemoradiotherapy for esophageal squamous cell carcinoma | No OS/DFS data |
| 70 | Tao H | 2017 | Phase II Trial of Intensity-Modulated Radiotherapy Concurrent With Chemotherapy for Postoperative Node-Positive Esophageal Squamous Cell Carcinoma | No OS/DFS data |
| 71 | Wang B | 2020 | Combination of Haemoglobin and Prognostic Nutritional Index Predicts the Prognosis of Postoperative Radiotherapy for Esophageal Squamous Cell Carcinoma | No OS/DFS data |
| 72 | Wang B | 2022 | Prognostic value of hemoglobin combined with Geriatric Nutritional Risk Index scores in patients undergoing postoperative radiotherapy for esophageal squamous cell carcinoma | No OS/DFS data |
| 73 | Wang LS | 1999 | Prognosis of esophageal squamous cell carcinoma: analysis of clinicopathological and biological factors | HR not available |
| 74 | Wang Q | 2020 | Postoperative adjuvant chemotherapy versus chemoradiotherapy for node-positive esophageal squamous cell carcinoma: a propensity score-matched analysis | No OS/DFS data |
| 75 | Wang X | 2016 | Recurrence pattern of squamous cell carcinoma in the midthoracic esophagus: implications for the clinical target volume design of postoperative radiotherapy | No OS/DFS data |
| 76 | Wang X | 2024 | Predictive and prognostic value of ACSL4 and GPX4 in patients with esophageal squamous cell carcinoma receiving post-operative radiotherapy | No OS/DFS data |
| 77 | Wang Y | 2015 | Factors on prognosis in patients of stage pT3N0M0 thoracic esophageal squamous cell carcinoma after two-field esophagectomy | HR not available |
| 78 | Wang Y | 2021 | Mapping of Cervical and Upper Mediastinal Lymph Node Recurrence for Guiding Clinical Target Delineation of Postoperative Radiotherapy in Thoracic Esophageal Squamous Cell Carcinoma | No OS/DFS data |
| 79 | Wang Y | 2016 | Patterns of recurrence in patients with stage pT3N0M0 thoracic esophageal squamous cell carcinoma after two-field esophagectomy | No English |
| 80 | Wang YX | 2017 | Patterns of recurrence in patients with stage thoracic esophageal squamous cell carcinoma after radical resection | No English |
| 81 | Wang Z | 2019 | Salvage lymphadenectomy for isolated cervical lymph node recurrence after curative resection of thoracic esophageal squamous cell carcinoma | No OS/DFS data |
| 82 | Wang ZW | 2014 | Postoperative chemoradiotherapy improves survival in esophageal squamous cell cancer with extracapsular lymph node extension | No OS/DFS data |
| 83 | Wang ZX | 2013 | Multimodality management of squamous cell carcinoma of thoracic esophagus | No English |
| 84 | Worni M | 2012 | Does surgery improve outcomes for esophageal squamous cell carcinoma? An analysis using the surveillance epidemiology and end results registry from 1998 to 2008 | HR not available |
| 85 | Xie X | 2025 | Postoperative adjuvant immunotherapy for pathological stage II-IVa esophageal squamous cell carcinoma after radical surgery does not improve disease-free recurrence rates | No OS/DFS data |
| 86 | Xu X | 2016 | Comparison and Prognostic Analysis of Adjuvant Radiotherapy versus Salvage Radiotherapy for Treatment of Radically Resected Locally Advanced Esophageal Squamous Cell Carcinoma | No OS/DFS data |
| 87 | Xu X | 2022 | Genetic variant of ADH1C for predicting survival in esophageal squamous cell cancer patients who underwent postoperative radiotherapy | No OS/DFS data |
| 88 | Xu ZC | 2023 | Pattern of relapse following three-field lymphadenectomy of esophageal carcinoma and related factors predictive of recurrence | No OS/DFS data |
| 89 | Yamamoto Y | 2025 | Comparison of outcomes between surgery and chemoradiotherapy after endoscopic resection for pT1a-MM with lymphovascular invasion or pT1b esophageal squamous cell carcinoma | No OS/DFS data |
| 90 | Yan L | 2018 | Sirtuin 2 (Sirt2) Expression Predicts Lymph Node Metastasis and Poor Overall Survival of Patients with Esophageal Squamous Cell Carcinoma | No OS/DFS data |
| 91 | Yang TY | 2024 | Survival Outcomes of Patients with Esophageal Cancer and Post-chemoradiotherapy Surgical T4b Disease: Is Palliative Resection Justified? | No OS/DFS data |
| 92 | Yao Q | 2023 | Low versus high dose of postoperative radiotherapy for locally advanced esophageal squamous cell carcinoma: a propensity score-matched analysis | No OS/DFS data |
| 93 | Yap WK | 2022 | Adjuvant Chemoradiotherapy Associated with Improved Overall Survival in Resected Esophageal Squamous Cell Carcinoma after Neoadjuvant Chemoradiotherapy in Intensity-Modulated Radiotherapy Era | No OS/DFS data |
| 94 | Ye L | 2021 | Para-aortic lymph node metastasis in lower Thoracic Esophageal Squamous Cell Carcinoma after Radical Esophagectomy: a CT-based atlas and its clinical implications for Adjuvant Radiotherapy | No OS/DFS data |
| 95 | Yu J | 2018 | Value of radiotherapy in addition to esophagectomy for stage II and III thoracic esophageal squamous cell carcinoma: Analysis of surveillance, epidemiology, and end results database | HR not available |
| 96 | Yu J | 2019 | Mapping patterns of metastatic lymph nodes for postoperative radiotherapy in thoracic esophageal squamous cell carcinoma: a recommendation for clinical target volume definition | No OS/DFS data |
| 97 | Yu S | 2020 | CREPT is a novel predictor of the response to adjuvant therapy or concurrent chemoradiotherapy in esophageal squamous cell carcinoma | No OS/DFS data |
| 98 | Zeng Y | 2019 | Difference in failure patterns of pT3-4N0-3M0 esophageal cancer treated by surgery vs surgery plus radiotherapy | Overlapping population |
| 99 | Zeng Y | 2024 | Postoperative tumor bed radiation versus T-shaped field radiation in the treatment of locally advanced thoracic esophageal squamous cell carcinoma: a phase IIb multicenter randomized controlled trial | No OS/DFS data |
| 100 | Zhang BH | 2013 | Clinical outcomes of basaloid squamous cell carcinoma of the esophagus: a retrospective analysis of 142 cases | No OS/DFS data |
| 101 | Zhang DK | 2009 | Clinical analysis of 22 cases of esophageal adenosquamous carcinoma | No English |
| 102 | Zhang W | 2014 | Epidermal growth factor receptor is a prognosis predictor in patients with esophageal squamous cell carcinoma | Overlapping population |
| 103 | Zhang W | 2015 | Postoperative intensity-modulated radiotherapy improved survival in lymph node-positive or stage III thoracic esophageal squamous cell carcinoma | Overlapping population |
| 104 | Zhang W | 2015 | Efficacy of intensity-modulated radiotherapy for resected thoracic esophageal squamous cell carcinoma | No OS/DFS data |
| 105 | Zhang W | 2023 | Prognostic significance and postoperative chemoradiotherapy guiding value of mean platelet volume for locally advanced esophageal squamous cell carcinoma patients | HR not available |
| 106 | Zhang X | 2018 | Recommendation for the definition of postoperative radiotherapy target volume based on a pooled analysis of patterns of failure after radical surgery among patients with thoracic esophageal squamous cell carcinoma | No OS/DFS data |
| 107 | Zhang X | 2019 | Adjuvant therapy for pathological T3N0M0 esophageal squamous cell carcinoma | HR not available |
| 108 | Zhang X | 2021 | The Prognosis and Feasibility of Extensive Clinical Target Volume in Postoperative Radiotherapy for Esophageal Squamous Cell Carcinoma: A Phase II Clinical Trial | No OS/DFS data |
| 109 | Zhang Y | 2017 | Treatment of esophageal cancer with radiation therapy - a pan-Chinese survey of radiation oncologists | No OS/DFS data |
| 110 | Zhang Y | 2024 | Survival risk stratification based on prognosis nomogram to identify patients with esophageal squamous cell carcinoma who may benefit from postoperative adjuvant therapy | No OS/DFS data |
| 111 | Zhang Z | 2020 | A retrospective study of postoperative radiotherapy for locally advanced esophageal squamous cell carcinoma | No OS/DFS data |
| 112 | Zhao Q | 2021 | Preliminary evaluation of postoperative radiotherapy with small T-shaped field in thoracic esophageal squamous cell carcinoma | No OS/DFS data |
| 113 | Zheng B | 2020 | Adjuvant chemoradiotherapy for patients with pathologic node-positive esophageal cancer following radical resection is associated with improved survival | No OS/DFS data |
| 114 | Zhou S | 2013 | Prognostic significance of XIAP and NF-κB expression in esophageal carcinoma with postoperative radiotherapy | No OS/DFS data |
| 115 | Zhu Y | 2016 | Postoperative radiation in esophageal squamous cell carcinoma and target volume delineation | No OS/DFS data |
| 116 | Zou B | 2020 | Radical esophagectomy for stage II and III thoracic esophageal squamous cell carcinoma followed by adjuvant radiotherapy with or without chemotherapy: Which is more beneficial? | No OS/DFS data |
| 117 | Chen KN | 2013 | Strengthen perioperative multimodality treatment to improve long-term outcomes of esophageal cancer | No OS/DFS data |
| 118 | Chen SB | 2012 | Clinical analysis of 36 cases of esophageal mucoepidermoid carcinoma | No OS/DFS data |
| 119 | Cao XF | 2010 | A prospective comparison between surgery alone and postoperative chemoradiotherapy for locally advanced esophageal squamous cell carcinoma | No English |
| 120 | Wang YX | 2016 | Patterns of recurrence in patients with stage pT2 N0-1M0 esophageal squamous cell carcinoma after radical resection | No English |
| 121 | Wang ZX | 2013 | Multimodality management of squamous cell carcinoma of thoracic esophagus | HR not available |
| 122 | Chen JQ | 2014 | Cervical lymph node metastasis classified as regional nodal staging in thoracic esophageal squamous cell carcinoma after radical esophagectomy and three-field lymph node dissection | Overlapping population |
| 123 | Zhang WC | 2014 | Epidermal growth factor receptor is a prognosis predictor in patients with esophageal squamous cell carcinoma | Overlapping population |
| 124 | Hsu PK | 2014 | Survival benefits of postoperative chemoradiation for lymph node-positive esophageal squamous cell carcinoma | Overlapping population |
| 125 | Zeng Y | 2019 | Difference in failure patterns of pT3-4N0-3M0 esophageal cancer treated by surgery vs surgery plus radiotherapy | Overlapping population |
| 126 | Hwang JY | 2015 | A Propensity-matched Analysis Comparing Survival After Esophagectomy Followed by Adjuvant Chemoradiation to Surgery Alone for Esophageal Squamous Cell Carcinoma | Overlapping population |
| 127 | Chen J | 2012 | Number and location of positive nodes, postoperative radiotherapy, and survival after esophagectomy with three-field lymph node dissection for thoracic esophageal squamous cell carcinoma | Overlapping population |
| 128 | Xiao ZF | 2003 | Value of radiotherapy after radical surgery for esophageal carcinoma: a report of 495 patients | Overlapping population |
| 129 | Wang LS | 1999 | Prognosis of esophageal squamous cell carcinoma: analysis of clinicopathological and biological factors | HR not available |
| 130 | Liang YH | 2023 | Lymph node ratio precisely predicts the benefit of postoperative radiotherapy in esophageal cancer: A retrospective cohort study | HR not available |
| 131 | Luo H | 2017 | Meta-analysis of survival benefit with postoperative chemoradiotherapy in patients of lymph node positive esophageal carcinoma | No OS/DFS data |
| 132 | Lee SJ | 2005 | Recurrence of squamous cell carcinoma of the oesophagus after curative surgery: rates and patterns on imaging studies correlated with tumour location and pathological stage. | No OS/DFS data |
| 133 | Guo XF | 2013 | Risk factors of recurrence and metastasis in pN1 stage squamous cell carcinoma of the thoracic esophagus after radical esophagectomy with lymphadenectomy | No English |
| 134 | Feng W | 2020 | Risk factors for tumor recurrence in patients with pT3N0M0 thoracic esophageal squamous cell carcinoma after esophagectomy | No OS/DFS data |
| 135 | Guo XF | 2015 | Clinical study on postoperative recurrence in patients with pN1 esophageal squamous cell carcinoma | No OS/DFS data |
| 136 | Lin HN | 2020 | A meta-analysis on surgery with or without postoperative radiotherapy to treat squamous cell esophageal carcinoma | No OS/DFS data |
